# The Healthy Activity Program (HAP), a lay counsellor-delivered brief psychological treatment for severe depression, in primary care in India: a randomised controlled trial

**DOI:** 10.1016/S0140-6736(16)31589-6

**Published:** 2017-01-14

**Authors:** Vikram Patel, Benedict Weobong, Helen A Weiss, Arpita Anand, Bhargav Bhat, Basavraj Katti, Sona Dimidjian, Ricardo Araya, Steve D Hollon, Michael King, Lakshmi Vijayakumar, A-La Park, David McDaid, Terry Wilson, Richard Velleman, Betty R Kirkwood, Christopher G Fairburn

**Affiliations:** a Sangath Centre, Socorro Village, Bardez-Goa, Goa, India; b Centre for Global Mental Health, Faculty of Epidemiology and Population Health, London School of Hygiene & Tropical Medicine, London, UK; c Medical Research Council Tropical Epidemiology Group; Faculty of Epidemiology and Population Health, London School of Hygiene & Tropical Medicine, London, UK; d Department of Psychology and Neuroscience, University of Colorado, Boulder, CO, USA; e Department of Psychology, Vanderbilt University, Nashville, TN, USA; f Department of Mental Health Sciences, University College London, London, UK; g Sneha, Voluntary Health Services, University of Melbourne, Melbourne, VIC, Australia; h Personal Social Services Research Unit, London School of Economics and Political Science, London, UK; i Department of Psychology, Rutgers School of Arts and Sciences, NJ, USA; j Department of Psychology, University of Bath, Bath, UK; k Department of Psychiatry, University of Oxford, Oxford, UK

## Abstract

**Background:**

Although structured psychological treatments are recommended as first-line interventions for depression, only a small fraction of people globally receive these treatments because of poor access in routine primary care. We assessed the effectiveness and cost-effectiveness of a brief psychological treatment (Healthy Activity Program [HAP]) for delivery by lay counsellors to patients with moderately severe to severe depression in primary health-care settings.

**Methods:**

In this randomised controlled trial, we recruited participants aged 18–65 years scoring more than 14 on the Patient Health Questionnaire 9 (PHQ-9) indicating moderately severe to severe depression from ten primary health centres in Goa, India. Pregnant women or patients who needed urgent medical attention or were unable to communicate clearly were not eligible. Participants were randomly allocated (1:1) to enhanced usual care (EUC) alone or EUC combined with HAP in randomly sized blocks (block size four to six [two to four for men]), stratified by primary health centre and sex, and allocation was concealed with use of sequential numbered opaque envelopes. Physicians providing EUC were masked. Primary outcomes were depression symptom severity on the Beck Depression Inventory version II and remission from depression (PHQ-9 score of <10) at 3 months in the intention-to-treat population, assessed by masked field researchers. Secondary outcomes were disability, days unable to work, behavioural activation, suicidal thoughts or attempts, intimate partner violence, and resource use and costs of illness. We assessed serious adverse events in the per-protocol population. This trial is registered with the ISRCTN registry, number ISRCTN95149997.

**Findings:**

Between Oct 28, 2013, and July 29, 2015, we enrolled and randomly allocated 495 participants (247 [50%] to the EUC plus HAP group [two of whom were subsequently excluded because of protocol violations] and 248 [50%] to the EUC alone group), of whom 466 (95%) completed the 3 month primary outcome assessment (230 [49%] in the EUC plus HAP group and 236 [51%] in the EUC alone group). Participants in the EUC plus HAP group had significantly lower symptom severity (Beck Depression Inventory version II in EUC plus HAP group 19·99 [SD 15·70] *vs* 27·52 [13·26] in EUC alone group; adjusted mean difference −7·57 [95% CI −10·27 to −4·86]; p<0·0001) and higher remission (147 [64%] of 230 had a PHQ-9 score of <10 in the HAP plus EUC group *vs* 91 [39%] of 236 in the EUC alone group; adjusted prevalence ratio 1·61 [1·34–1·93]) than did those in the EUC alone group. EUC plus HAP showed better results than did EUC alone for the secondary outcomes of disability (adjusted mean difference −2·73 [–4·39 to −1·06]; p=0·001), days out of work (−2·29 [–3·84 to −0·73]; p=0·004), intimate partner physical violence in women (0·53 [0·29–0·96]; p=0·04), behavioural activation (2·17 [1·34–3·00]; p<0·0001), and suicidal thoughts or attempts (0·61 [0·45–0·83]; p=0·001). The incremental cost per quality-adjusted life-year gained was $9333 (95% CI 3862–28 169; 2015 international dollars), with an 87% chance of being cost-effective in the study setting. Serious adverse events were infrequent and similar between groups (nine [4%] in the EUC plus HAP group *vs* ten [4%] in the EUC alone group; p=1·00).

**Interpretation:**

HAP delivered by lay counsellors plus EUC was better than EUC alone was for patients with moderately severe to severe depression in routine primary care in Goa, India. HAP was readily accepted by this previously untreated population and was cost-effective in this setting. HAP could be a key strategy to reduce the treatment gap for depressive disorders, the leading mental health disorder worldwide.

**Funding:**

Wellcome Trust.

## Introduction

Depression is the leading mental health cause of the global burden of disease,^1^ with a global prevalence of 4·7%.^2^ Depression substantially impairs quality of life, social functioning, and workforce participation among people with the disease, their family members, and their communities,^3^, ^4^ with an annual global cost attributable to depression estimated at US$1·15 trillion.^4^ Certain psychological treatments can be as effective as antidepressant medications, with higher retention and better enduring effects,^5^ and they are recommended as first-line interventions by WHO's Mental Health Gap Action Programme (mhGAP).^6^ However, most depressed people who live in low-income and middle-income countries do not have access to psychological treatments. Authors of reviews have shown that this treatment gap exceeds 90% in India and China^7^ and often exceeds 50%,^8^ even in highly resourced settings.

**Research in context**

**Evidence before this study**

Our review began with the evidence synthesised by WHO's Mental Health Gap Action Programme guidelines for mental disorders in non-specialised health-care settings. We supplemented this evidence with searches of PubMed and PsycINFO from Jan 1, 2009, to Jan 1, 2011, with no language restrictions, as well as hand searching reference lists of selected papers, contacting key informants, and visiting key libraries in the region. Structured psychological treatments based on cognitive behavioural or interpersonal theories are recommended as first-line interventions by WHO's Mental Health Gap Action Programme for moderate to severe depression. Simpler versions of these treatments, such as behavioural activation, are as effective as are more elaborate versions, such as cognitive behaviour therapy. However, the existing evidence has limited generalisability to many low-income and middle-income countries where both supply side (low availability of mental health professionals) and demand side (low levels of mental health literacy) barriers lead to large treatment gaps.

**Added value of this study**

This study is the first report of findings from any low-income and middle-income country assessing the effectiveness and cost-effectiveness of a psychological treatment for moderately severe to severe depression in primary care. A brief (six to eight sessions) psychological treatment (the Healthy Activity Program), based on behavioural activation, delivered by lay counsellors, was better than was enhanced usual care according to all prespecified primary clinical and secondary social and functional outcomes. HAP was readily accepted by this previously untreated population and was cost-effective in this setting.

**Implications of all the available evidence**

Brief psychological treatments, based on behavioural activation, are acceptable, feasible, and cost-effective for management of moderate to severe depression, even when delivered by non-specialist health workers in routine health-care settings in previously untreated populations. Such treatments should be scaled up as a key strategy to address depressive disorders, the leading mental health disorder worldwide.

Most of the evidence supporting psychological treatments comes from specialist settings in high-income countries and the generalisability of the findings to low-income and middle-income countries can be questioned. Contextual factors, such as variations in explanatory models, ways of coping with distress, little access to specialist services, and socioeconomic barriers such as literacy should be considered.^9^, ^10^ However, evidence is growing for the effectiveness of contextually sensitive psychological treatments that have been adapted to the local context and delivered by appropriately trained and supervised lay health workers in primary care and community settings.^11^ Identification of effective psychological treatments that can be delivered in this way has been ranked among the leading research priorities for global mental health.^12^

The goal of the Program for Effective Mental Health Interventions in Under-Resourced Health Systems (PREMIUM) was to develop and assess scalable psychological treatments that are culturally appropriate, affordable, and feasible for delivery by non-specialist health workers and apply these treatments to the two leading mental health disorders: moderately severe to severe depression (the Healthy Activity Program [HAP]) and harmful drinking (Counselling for Alcohol Problems [CAP]).^13^, ^14^, ^15^ In this Article, we describe the results of a trial assessing the effectiveness and cost-effectiveness of HAP,^13^ a brief psychological treatment adapted from behavioural activation, an empirically supported psychological treatment recommended by WHO.^16^, ^17^ A core feature of PREMIUM is delivery of both treatments by the same lay counsellors in routine primary care settings, thus reproducing the way that they would be used in clinical practice. The results of the trial assessing the CAP treatment for harmful drinking are published separately.^18^ The two trials of HAP and CAP were done concurrently in the same primary health centres (PHCs) and over the same period of time, with the same counsellors delivering both treatments according to trial allocations of participants.

## Methods

### Study design and participants

In this randomised controlled trial, we recruited participants aged 18–65 years who had a probable diagnosis of moderately severe to severe depression ascertained with a Patient Health Questionnaire 9 (PHQ-9) score of more than 14, a cutpoint previously validated in the study setting,^19^ in ten primary health centres in Goa, a state on the west coast of India. The publicly funded PHCs are the first option for financially disadvantaged people. Pregnant women and patients who needed urgent medical attention or were unable to communicate clearly were not eligible. These criteria were established and applied by trained health assistants at the PHC.

The trial protocol^20^ was approved by the Trial Steering Committee, and ethical approval for the conduct of the trial was obtained from the Institutional Review Boards of the London School of Hygiene & Tropical Medicine, Sangath (the implementing institution in India), and the Indian Council of Medical Research. Written (or witnessed, if the participant was illiterate) informed consent was mandatory for enrolment. We audiotaped all consent procedures, with patients' approvals.

### Randomisation and masking

We randomly allocated participants (1:1) to HAP plus enhanced usual care (EUC) or EUC alone. An independent statistician generated a randomisation list in randomly sized blocks (block size four to six [two to four for men because we anticipated relatively fewer men on the basis of the epidemiology of the prevalence of depression and did not want imbalance between groups]), stratified by PHC and sex. Assignments were sealed in sequential numbered opaque envelopes by independent support staff that were opened as each consenting eligible patient was enrolled^21^ by trained health assistants. Physicians providing EUC were masked to allocation status, as were the independent assessors who did outcome assessments, and these people had no contact with the PHCs or other team members. All authors, apart from the data manager (BB), were masked until the trial results were unmasked in the presence of the Trial Steering Committee and Data Safety and Monitoring Committee on March 7, 2016. Instances of unmasking of outcome assessors in the HAP group will be summarised on the basis of overall prevalence and the exact point during the interview that the interviewer was unmasked.

### Procedures

We interviewed patients who gave informed consent to collect data for sociodemographic factors and potential moderators of treatment effect: sex, duration of illness, and expectations for treatment.^22^ We reduced the number of potential moderators (following approval from the Trial Steering Committee and data and safety monitoring board) listed in the trial protocol to align our analyses with what is commonly reported in the literature regarding moderators in depression treatment trials.^22^, ^23^ We recorded all intake interviews and randomly selected (using a random selection strategy statified by health assistant) a subset for review by a supervisor for quality assurance.

In the enhanced usual care group, usual care and treatment provided by the PHC physician was enhanced by provision of screening results to both patient and physician and use of a contextualised version of the mhGAP guidelines (a manual containing the specific mhGAP guidelines for primary care physicians treating depression),^16^ including when and where to refer for psychiatric care. In the HAP group, participants received EUC plus HAP,^13^ a manualised psychological treatment based on behavioural activation that includes the following strategies: psychoeducation, behavioural assessment, activity monitoring, activity structuring and scheduling, activation of social networks, and problem solving. Additional strategies used in response to specific needs consisted of behavioural strategies to improve interpersonal communication skills and decrease rumination, advice regarding sleep problems and tobacco cessation, and relaxation training.

On the basis of extensive formative and pilot research,^13^, ^24^ we made various adaptations to enhance the contextual acceptability and feasibility of the treatment, such as inclusion of home-based delivery, use of pictorial patient resource materials, strategies to encourage involvement of a significant other in treatment, and sharing of supervision of lay counsellors with peer groups. Additionally, we condensed delivery of the core intervention strategies from the normal 20–24-session format^25^ into a six-session to eight-session protocol. We delivered HAP in an individual format, each lasting 30–40 min, with initial sessions at weekly intervals. We typically conducted sessions face-to-face, at the PHC or patient's home, but used telephone sessions when necessary. The beginning phase focused on orientation to treatment, a multisession middle phase focused on teaching of core intervention strategies, and a late phase focused on review of gains and termination. The middle phase could be extended by up to two additional sessions for patients who did not show sufficient improvement (consistently high PHQ-9 scores and absence of activation), allowing a maximum of eight sessions. Patients who did not respond could be referred for specialist care. We considered patients who met treatment goals or completed the maximum number of sessions or were referred to specialists to have a planned discharge; we considered all other patients to have an unplanned discharge.

A detailed description of counsellor selection and training is provided elsewhere.^19^, ^26^ An international expert in behavioural activation (SD) trained and provided ongoing supervision for five local specialists, who in turn provided onsite training and supervision for lay counsellors. Training of lay counsellors involved a 3 week participatory workshop covering both HAP and CAP treatments, followed by an internship phase of 6 months, in which trainee counsellors delivered the treatment to eligible patients in primary health-care clinics, combined with peer-led group supervision as the trainees gained experience in delivery of the treatment.^26^ 11 counsellors who met competency standards as assessed by standardised roleplays and therapy quality measures participated in the trial. They received weekly peer-led supervision in groups of four to six that involved rating of a randomly selected (using a random selection strategy stratified by counsellor and phase of session) 10% of recorded sessions on the HAP Therapy Quality Scale (TQS)^26^ and individual supervision twice monthly. HAP delivery costs included patient contact and counsellor training, supervision, and salary.

We assessed treatment fidelity via treatment completion, maintained by counsellors in electronic clinical records, HAP TQS scores from peer and expert ratings of audio recordings of sessions during weekly group supervision, and therapy quality of a randomly selected (using a random selection strategy stratified by counsellor and phase of session) 10% of all sessions rated by an independent expert who was not involved in the trial but had previously led development of HAP.^13^

### Outcomes

The two primary outcomes were depression severity assessed by the modified (dropping the item related to sex for cultural reasons) Beck Depression Inventory version II (BDI-II) and remission from depression as defined by a PHQ-9 score of less than 10, both assessed 3 months after enrolment. Secondary outcomes were disability on the WHO Disability Assessment Schedule II and total days unable to work in the previous month, behavioural activation on the five-item abbreviated Activation Scale based on the Behavioural Activation for Depression Scale-Short Form, suicidal thoughts or attempts in the past 3 months, intimate partner violence (not a prespecified hypothesis), and resource use and costs of illness estimated from the Client Service Receipt Inventory.^27^ We assessed behavioural activation concurrently with the primary outcome, precluding drawing of any causal inference. Concurrent assessment of mediator and outcome provides only a very weak test of mediation and we are hesitant to report findings in that regard since we consider them potentially misleading. Instead, we have reframed behavioural activation as a putative target of the treatment rather than an outcome in its own right. We will be examining the potential mediating role of behavioural activation on the effect of intervention at 12 months separately. All adaptations to outcomes, including the decision to drop the item enquiring about sexual drive (because of cultural sensitivity considerations), were finalised and approved by the Trial Steering Committee and the Data and Safety Monitoring Committee before the start of the trial. We collected data for serious adverse events, defined as deaths, suicide attempts, and unplanned admissions to hospital from any cause.

### Statistical analysis

Assuming an intracluster correlation between clinics of 0·04, with one counsellor per PHC at any one time, a loss to follow-up of 15% over 3 months, and a 1:1 allocation ratio, we aimed to recruit 500 participants to detect the hypothesised effects (a standardised mean difference of 0·42), with 90% power for the primary continuous outcome of depression severity and 92% power to detect a recovery of 65% in the HAP group for our primary binary outcome of depression remission. We took into account the design effect of 1·28 in the sample size estimation.

Analyses were on an intention-to-treat basis with use of multiple imputation for those with missing outcome data. We assessed serious adverse events in the per-protocol population. All models adjusted for PHC as a fixed effect to allow for within-PHC clustering and adjusted for baseline PHQ-9 scores. For continuous outcomes, we estimated intervention effects using linear regression and reported them as adjusted mean differences (AMDs) and effect sizes, with 95% CIs. For binary outcomes, we reported intervention effects as adjusted prevalence ratios (aPRs) estimated from logistic regression using the marginal standardisation technique for prevalence ratios and the δ method for the 95% CIs.^28^ Although our original plan was to have one counsellor per PHC, in practice, 11 counsellors delivered the intervention over the ten PHCs. Of these, six worked in one PHC only, and the remaining five worked in two PHCs where caseloads were higher. Thus, we carried out a sensitivity analysis as a multilevel model with both PHC and counsellor included as random effects. Sensitivity analyses also included complete case analyses. We did Complier's Average Causal Effect analyses to estimate the effect of completed treatment as originally intended on participants.^29^

Results are described in terms of strength of evidence rather than statistical significance^30^ and, hence, we did not adjust p values for multiple comparisons. We did economic assessments from health-care system and societal perspectives. We derived quality-adjusted life-year (QALY) scores using an approach previously used in India.^31^ We bootstrapped incremental cost-effectiveness ratios to derive 95% CIs. We explored statistical uncertainty around the incremental cost-effectiveness ratios through cost-effectiveness acceptability curves showing the likelihood that HAP would be cost-effective at different levels of willingness to pay. We did post-hoc analyses of remission as defined by a PHQ-9 score of less than 5 or a 50% reduction in score as a stringent indicator of remission. We did all statistical analyses using Excel 2016, SPSS 21, and Stata 14. All costs are presented in 2015 international dollars. A Data and Safety Monitoring Committee oversaw the trial. This trial is registered with the ISRCTN registry, number ISRCTN95149997.

### Role of the funding source

The funder of the study had no role in study design, data collection, data analysis, or writing of the report. VP, HAW, BW, BB, DM, and A-LP had full access to all the data in the study. VP and BW had final responsibility for the decision to submit for publication.

## Results

**Figure 1:**
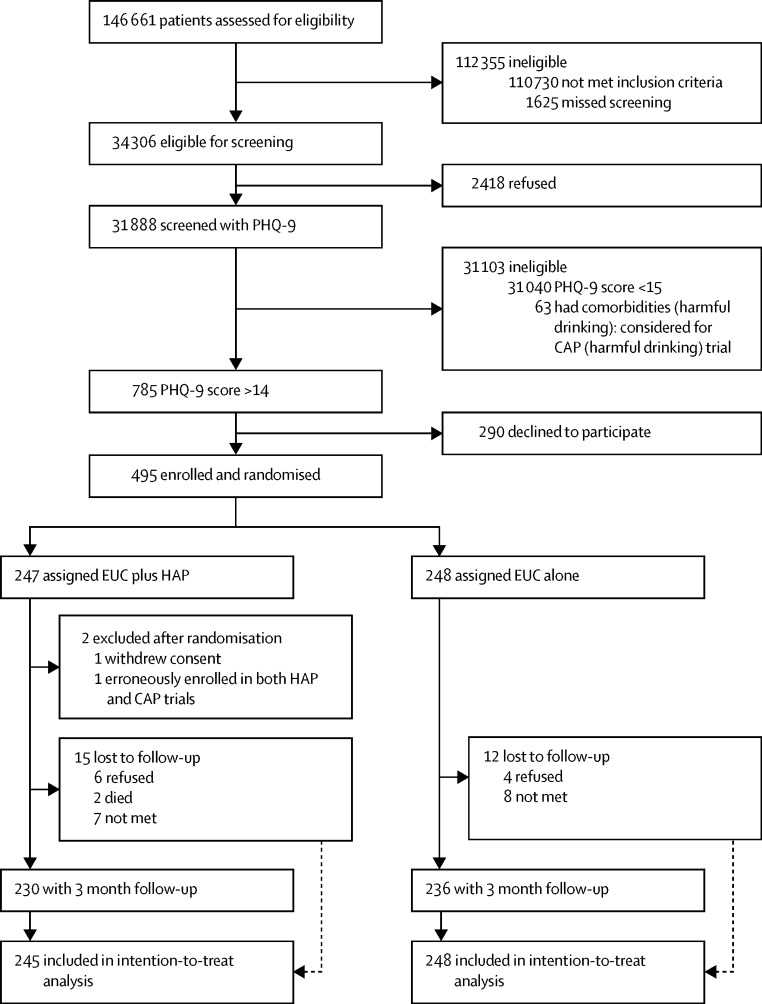
Trial profile CAP=Counselling for Alcohol Problems. EUC=enhanced usual care. HAP=Healthy Activity Program. PHQ-9=Patient Health Questionnaire 9.

Between Oct 28, 2013, and July 29, 2015, 34 306 (23%) of the 146 661 PHC attendees assessed met eligibility criteria (figure 1). 31 888 (93%) of these patients were screened for depression using PHQ-9 (18 740 [59%] women and 13 148 [41%] men) and 785 (2%) met criteria for inclusion in the trial (615 [78%] women and 170 [22%] men), of whom 495 (63%) consented to participate and were enrolled (379 [77%] women and 116 [23%] men). 248 patients were randomly allocated to EUC and 247 were randomly allocated to EUC plus HAP, two of whom were subsequently excluded from the EUC plus HAP group (one withdrew consent and the other was erroneously enrolled in both CAP and HAP trials), leaving a total of 245 patients given EUC plus HAP.

2480 (8%) of 31 888 patients screened had moderate to severe depression, as defined by a PHQ-9 score of more than 10, which is within the range of prevalence of depression reported in the WHO multinational study of common mental disorders in general health care.^32^ Baseline characteristics were similar by group (table 1). The modal patient was a married woman in her early forties with moderately severe depression, with a primary school education, and not employed outside the home. Participants had similar baseline characteristics to those who declined (appendix), and the most common reason for refusal to participate in the trial was time constraints (118 [41%]). 466 (94%) participants were seen at the 3 month endpoint (236 [51%] in the EUC group and 230 [49%] in the EUC plus HAP group), with a mean interval of 14·8 weeks (95% CI 14·6–15·2). Those lost to follow-up tended to be younger and more likely to be single than were those not lost to follow-up (appendix). The trial was completed on Aug 30, 2016, when the 12 month outcome assessment ended.

**Figure 2:**
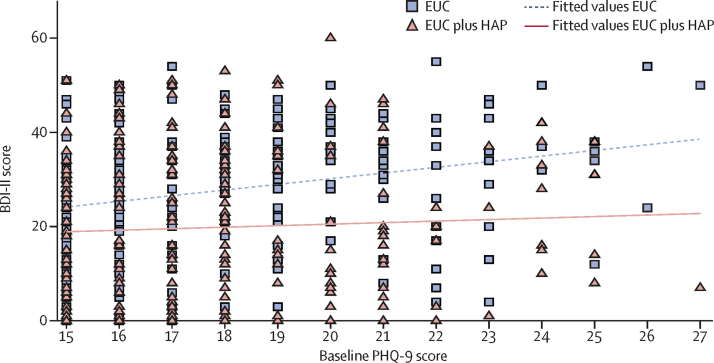
Effect of baseline PHQ-9 score on depression severity according to BDI-II score at 3 months BDI=Beck Depression Inventory. EUC=enhanced usual care. HAP=Healthy Activity Program. PHQ=Patient Health Questionnaire.

We noted strong evidence of an intervention effect on depression symptom severity. Mean BDI-II score was 27·52 (SD 13·26) in the EUC group and 19·99 (15·70) in the EUC plus HAP group (AMD −7·57 [95% CI −10·27 to −4·86]; p<0·0001; table 2), with an effect size of 0·48 (95% CI 0·30–0·66). We noted some evidence of a treatment interaction with severity, with a larger effect of EUC plus HAP than for EUC alone among patients with more severe depression at baseline (PHQ-9 score of 20 or more) than among those who were less severe (p value for interaction 0·05); differences were significant within each subset (high severity: mean BDI-II score in EUC group 32·71 [SD 12·75] *vs* 20·16 [15·45] in EUC plus HAP group, AMD −12·15 [95% CI −17·53 to −6·77], p<0·0001; moderate severity: 25·80 [13·19] *vs* 20·16 [15·82], AMD −5·99 [–9·14 to −2·85]; p<0·0001; figure 2). We obtained similar results fitting HAP counsellor as a random effect and using complete case analyses (appendix). We noted no evidence of moderation by sex, chronicity, or expectation (appendix).

HAP also had a strong intervention effect on depression remission (PHQ-9 score of <10), with 147 (64%) of 230 participants meeting criteria in the EUC plus HAP group compared with 91 (39%) of 236 in the EUC alone group (aPR 1·61 [95% CI 1·34–1·93]; adjusted risk difference 24·0% [15·4–32·6]; p<0·0001). This finding yields a number needed to treat of 4·15 (3·07–6·45). The Complier's Average Causal Effect analysis for the effect of having a planned discharge found a prevalence ratio of 2·16 (1·68–2·78) associated with remission among patients who completed HAP. HAP remained better when remission was defined more stringently (PHQ score of <5: aPR 2·34 [1·75–3·14]) or as an at least 50% reduction in PHQ score (aPR 1·82 [1·51–2·19]).

The effect of EUC plus HAP was better than that of EUC for all secondary outcomes (WHO Disability Assessment Schedule II effect size 0·13; days unable to work effect size 0·20) and the putative target of behavioural activation (Behavioural Activation for Depression Scale effect size −0·39), except for intimate partner psychological violence among women or any form of violence among men (table 2). Serious adverse events and prescription of antidepressant medications were infrequent and did not differ between treatments (any serious adverse event nine [4%] in the HAP plus EUC group *vs* ten [4%] in the EUC alone group, p=1·00; deaths two [1%] *vs* none, p=0·24; suicide attempts four [2%] *vs* three [1%], p=0·72; unplanned admissions to hospital three [1%] *vs s* even [3%], p=0·34); appendix).

The intraclass correlation of BDI-II within PHCs was 0·005. The internal consistency (Cronbach's α) observed in this study was 0·86 for PHQ-9 and 0·91 for BDI-II. Of the 245 participants in the HAP group, 169 (69%) had a planned discharge, of whom seven (4%) were referred for specialist care. Their median number of sessions was six (IQR five to seven). 61 (25%) received more than the optimal dose of six sessions. Patients with an unplanned discharge were likely to stop attending early (median one session [IQR none to two]). Of the total 1181 sessions delivered, 1133 (96%) were face-to-face. Although 173 (77%) of the 226 first sessions (typically on the day when patients were enrolled) took place in the PHC, 522 (91%) of the 575 subsequent sessions were delivered at home. 51 (21%) of participants had a significant other involved in at least one session. The mean session duration was 40·2 min (95% CI 39·1–41·4). Mean TQS score was 2·58 (2·53–2·64) on the basis of peer supervisor ratings (n=186), similar to expert supervisor ratings (n=186; mean 2·55 [2·47–2·62]) and the mean score of the independent rater (n=100; mean 2·76 [2·69–2·82]), indicating good to average therapy quality. The most frequently endorsed HAP elements by the counsellor at the end of treatment were activation (178 [73%]), followed by problem solving (62 [25%]). 31 (13%) of 245 investigators were unmasked, with 16 (7%) unmasked before the primary outcome assessment.

From the health system perspective, the total health-care cost per person, including the intervention cost, was significantly higher in the EUC plus HAP group than in the EUC alone group, with significantly better QALY scores (table 3). Excluding the cost of intervention, the use and thus cost of other health-care service costs was not different between groups. Health system planners will need to consider whether or not the additional budgetary costs of investment in HAP are worth the health improvements gained. One aid to decision making is assessment of cost-effectiveness; an incremental cost per QALY gained of less than gross domestic product per capita has been considered to be highly cost-effective.^33^ HAP has a favourable cost per QALY gained (table 4) as GDP per capita in the state of Goa in 2015 was $16 060.^34^

**Figure 3:**
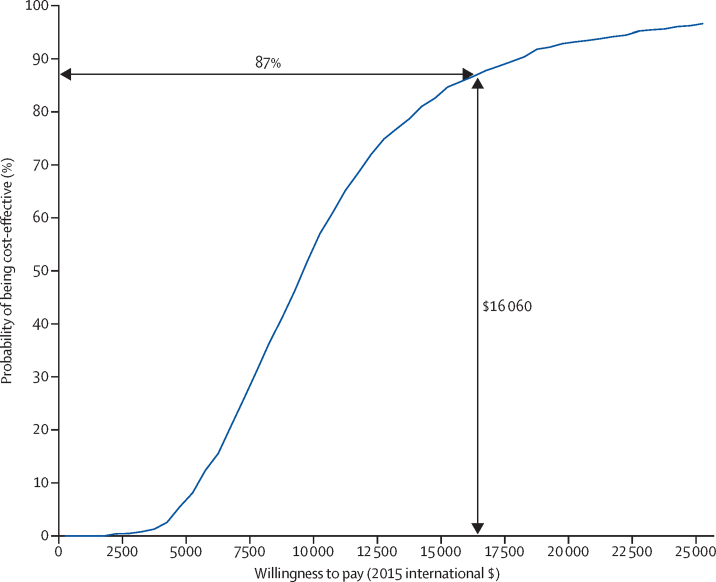
Cost-effectiveness acceptability curve: willingness to pay per quality-adjusted life-year gained from the Healthy Activity Program from a health system perspective

Figure 3 provides a cost-effectiveness acceptability curve, indicating that HAP has an 87% chance of being considered cost-effective from a health system perspective in the state of Goa. No consensus exists on the cost-effectiveness thresholds per additional remission at 3 months on the PHQ-9 scale, but as table 4 shows, the incremental costs are modest; if society is willing to pay up to the equivalent of 1 month's wages in Goa for an unskilled manual worker per remission ($415),^35^ HAP has a 99% chance of being cost-effective (appendix). The cost per BDI-II point improvement was $6, approximately one third of the daily wages of an unskilled worker in Goa. From a societal perspective, productivity losses due to days unable to work and work cutback are significantly lower in the EUC plus HAP group than in the EUC alone group (table 3), which means that the overall costs of HAP are no longer significantly different to those in the EUC alone group. This finding means that HAP then has a 98% chance of being cost-effective and a 42% chance of being cost saving—ie, having lower costs and better outcomes than has EUC alone, with similar results for remission and BDI-II outcomes (appendix).

## Discussion

HAP as delivered by non-specialist health workers in routine primary care settings is effective in treatment of moderately severe and severe depression. HAP produced a moderate effect on depressive symptoms, an almost two-thirds increase in remission, and small effects on secondary outcomes related to functional impairment after only six to eight sessions of treatment. Baseline severity slightly moderated the effects of treatment; as in other trials,^36^ the magnitude of the effect was largest among participants with severe depression, but unlike those other trials, the effect is also present among those less severely depressed. The economic analysis per QALY gained indicates that HAP is cost-effective; when wide societal effects on productivity are considered, the economic case is further strengthened, with a high probability that the intervention could be cost saving. The numbers of serious adverse events observed were small and consistent with our observations in a previous trial^37^ in primary care with patients with common mental disorders.

The effect of HAP on secondary outcomes is important for multiple reasons. Functional impairment can persist after a major depressive episode and represents an important target of treatment. The pragmatic emphasis on behaviour change in HAP might be valuable to guide individuals with depression to improve functional capacity and capacity to work. Moreover, most patients with depression are women and the association between sex disadvantage and depression among women has been widely documented.^38^ Women who received HAP were nearly 50% less likely to report intimate partner physical violence at the end of treatment than were women in usual care. The focus of HAP on improvement of life context and problem solving directly targets many of the components of sex disadvantage that have been associated with depression among women, including those that are specific to the family and interpersonal context.^38^ This finding should be interpreted with caution and examined further in future trials. The economic case will be strengthened if differences in health service use and productivity are sustained over time. The marginal costs of HAP delivery would also fall once a pool of trained lay counsellors has been established that can continue to provide services. Moreover, the effect of HAP on depression outcomes was similar to that of other studies of behavioural activation and cognitive and behavioural interventions delivered by specialist providers over more sessions than in this study.^39^

The study has certain limitations. First, even though HAP was better than what is usually done, about a third of participants remained depressed even after treatment. Our emphasis on the brevity of the intervention could possibly have resulted in this less than optimal absolute effect. Still, we believe at the very least that HAP would be a suitable candidate for the initial intervention in a stepped care system of treatment delivery for severe depression.^40^ Second, our two measures of depression were somewhat discordant at the end of treatment; thus, although BDI-II scores suggested that patients ended treatment at the low end of the moderate range of severity (on the basis of the norms used in trials in high-income countries),^41^ PHQ-9 indicated only mild residual symptoms. We have more confidence in PHQ-9 than in BDI-II in this regard as it has been used extensively cross-culturally and validated in the study setting.^19^ Furthermore, we caution against application of the existing norms to our study population as BDI-II was administered in an interview format rather than as self-report. Third, diagnostic interviews were not carried out at baseline, although PHQ-9 is widely used to define case-level morbidity in trials and, importantly, we used locally validated cutoffs in this study.^19^ Finally, the results in this study are restricted to the primary endpoint outcomes at 3 months for which our interest lies in the response and remission to our treatment of participants with moderately severe to severe depression. We intend to assess the sustainability of these outcomes, including recovery from depression, at a 12 month follow-up.

The findings of this trial, along with the companion trial^18^ assessing CAP treatment, represent a substantial step in global mental health for several reasons. First, the interventions are brief, delivered by lay people and provided to primary health-care attenders with almost no exclusion criteria, thus enhancing their generalisability. Second, treatment was delivered by the same counsellors who concurrently delivered treatment for alcohol problems. We have thus shown the potential real-world value of our study to address the most common mental health and substance use disorders encountered in primary care by a single counsellor. However, the real-world responsibilities of such a counsellor could potentially extend to other mental disorders, such as psychoses, and future implementation research would need to address how the PREMIUM treatments can be integrated with these broad roles. Third, the treatment was adapted from behavioural activation, a treatment that has a strong theoretical and empirical evidence base across multiple countries and patient populations.^17^ Findings from the COBRA trial^42^ from the UK have further substantiated the equivalence of this simpler psychological treatment with more elaborate treatments such as cognitive behavioural therapy. Fourth, the conduct and analyses of the trials were carried out in adherence with the protocol and the trial indicators testify to high levels of internal validity. Finally, the target population has very low levels of awareness of depression as a mental health condition and virtually all participants primarily present for somatic concerns.^43^ Few, if any, would have had a previous diagnosis of depression or received a psychological treatment or antidepressants, as evidenced by the low number of prescriptions, even after the diagnosis was conveyed to the primary care physician.^44^ Ratings of therapy quality, both independent and by supervisors, and the high levels of treatment completion testify to the acceptability and feasibility of this non-specialist-delivered treatment, even in this previously untreated population.

This particular trial represents, to our knowledge, the first ever publication from any low-income and middle-income country assessing the effectiveness and cost-effectiveness of a psychological treatment for moderately severe to severe depression in primary care. Further research should focus on replication of our findings and dissemination of HAP in routine care settings in diverse contexts. Our dissemination efforts for HAP include launching of an online platform for those interested to learn the treatment^45^, ^46^ and collaborations to reverse engineer the treatment by assessing its effects in high-income settings, such as in the USA. A documentary about the two PREMIUM trials can also be accessed online.^47^ We anticipate that these features will be key to the long-term goal of the evidence generated by PREMIUM contributing to closing of the treatment gap for mental disorders worldwide.

## Figures and Tables

**Table 1:** Baseline characteristics

		**EUC plus HAP (n=245)**	**EUC alone(n=248)**
Age (years)		42·4 (12·1)	42·6 (12·0)
Female sex		188 (76%)	191 (77%)
Marital status			
	Married	166 (68%)	171 (69%)
	Single	28 (11%)	29 (12%)
	Separated or divorced	3 (1%)	1 (0·4%)
	Widowed	48 (19%)	47 (19%)
Educational status			
	None	75 (31%)	55 (22%)
	Primary	114 (46%)	135 (54%)
	Secondary	38 (16%)	40 (16%)
	Higher secondary	13 (5%)	11 (4%)
	Graduate or above	5 (2%)	7 (3%)
Occupation			
	Unemployed*	152 (62%)	140 (56%)
	Unskilled manual labour	77 (31%)	97 (39%)
	Skilled manual labour	3 (1%)	4 (2%)
	Clerical and professional	13 (5%)	7 (3%)
Patient's expectation of usefulness of counselling			
	Not useful	0	1 (<1%)
	A little or somewhat useful	115 (47%)	111 (45%)
	Moderately useful	66 (27%)	50 (20%)
	Very useful	64 (26%)	86 (35%)
Chronicity of symptoms (weeks)		16 (4–48)	12 (4–36)
PHQ-9 score		17·9 (2·8)	17·9 (2·6)
PHQ category			
	Score 15–19 (moderately severe)	185 (76%)	187 (75%)
	Score 20–27 (severe)	60 (24%)	61 (25%)

Data are mean (SD), n (%), or median (IQR). EUC=Enhanced usual care. PHQ-9=Patient Health Questionnaire 9.

* Includes students and people not working outside of the home.

**Table 2:** Primary and secondary outcomes

	EUC plus HAP (n=245)*	EUC alone (n=248)*	**Adjusted mean difference (95% CI)**†	**p value**
**Primary outcomes**				
BDI-II score at 3 months	19·99 (15·70)	27·52 (13·26)	−7·57 (−10·27 to −4·86)	<0·0001
Depression remission at 3 months	147/230 (64%)	91/236 (39%)	1·61 (1·34 to 1·93)	<0·0001
**Secondary outcomes**				
Disability score	9·12 (9·34)	11·79 (8·99)	−2·73 (−4·39 to −1·06)	0·001
Days unable to work	4·97 (7·71)	7·21 (9·38)	−2·29 (−3·84 to −0·73)	0·004
Behavioural activation score	12·05 (4·76)	9·88 (4·36)	2·17 (1·34 to 3·00)	<0·0001
Suicidal thoughts or attempts‡	48/230 (21%)	82/236 (35%)	0·61 (0·45 to 0·83)	0·001
Intimate partner physical violence (women)§	13/112 (12%)	26/120 (22%)	0·53 (0·29 to 0·96)	0·04
Intimate partner psychological or emotional violence (women)§	33/112 (29%)	44/120 (37%)	0·76 (0·53 to 1·10)	0·15
Intimate partner physical violence (men)§	0/37	3/40 (8%)	0·00	0·24¶
Intimate partner psychological or emotional violence (men)§	6/37 (16%)	8/40 (20%)	0·67 (0·27 to 1·71)	0·41

Data are mean (SD) or n/N (%). EUC=enhanced usual care. HAP=Healthy Activity Program. BDI=Beck Depression Inventory.

* Among those with observed data at 3 months.

† Including imputed outcome data for those with missing data.

‡ We assessed suicidal thoughts over the past 2 weeks through the relevant Patient Health Questionnaire 9 item, whereas we assessed suicide attempts over the 3 month period since enrolment. Seven participants reported suicide attempts (three in the EUC group and four in the EUC plus HAP group). All of these participants also reported suicidal thoughts.

§ Among married participants.

¶ Because of sparse data, the p value for physical violence in men is using Fisher's exact test on complete case data.

**Table 3:** Costs per person and QALYs gained (2015 international dollars)

	**EUC plus HAP (n=245)**	**EUC alone (n=248)**	**Mean difference (95% CI)**	**p value**
**Health system costs ($)**				
PHC doctor consultations	$13 (21)	$15 (23)	−$2 (−6 to 2)	0·40
Hospital doctor consultations	$13 (73)	$13 (51)	$0 (−11 to 11)	0·98
Hospital admissions	$8 (62)	$22 (100)	−$14 (−28 to 2)	0·08
Laboratory tests	$6 (20)	$9 (36)	−$3 (−8 to 2)	0·24
Medicines	$6 (15)	$9 (25)	−$3 (7 to 1)	0·08
Total public health-care costs	$47 (117)	$67 (132)	−$20 (−40 to 4)	0·07
HAP intervention	$66 (55)	$0	$66 (59 to 73)	<0·0001
**Productivity costs ($)**				
Time costs to service users and families	$52 (62)	$40 (55)	$12 (1 to 22)	0·03
Productivity losses	$87 (116)	$139 (141)	−$52 (−75 to −29)	<0·0001
**Total costs ($)**				
Health system perspective	$113 (125)	$67 (132)	$46 (22 to 68)	<0·0001
Societal perspective	$251 (229)	$246 (244)	$5 (−37 to 47)	0·83
**Cost-effectiveness analyses**				
QALYs gained	0·209 (0·018)	0·204 (0·018)	0·005 (0·002 to 0·008)	0·008

Data are mean (SD). EUC=enhanced usual care. HAP=Healthy Activity Program. PHC=public health centre. QALY=quality-adjusted life-year.

**Table 4:** Cost-effectiveness analyses from health system and societal perspectives (2015 international dollars)

	**Health system perspective**	**Societal perspective**
Cost per QALY gained at 3 months ($)	$9333 (3862 to 28 169)	$957 (−6145 to 14 418)
Cost per remission at 3 months ($)	$181 (87 to 335)	$19 (−133 to 229)
Cost per BDI point improvement at 3 months ($)	$6 (3 to 12)	$1 (−5 to 8)

Data are mean (95% CI). BDI=Beck Depression Inventory.
