# Supplementary material for: The Healthy Activity Program (HAP), a lay counsellor-delivered brief psychological treatment for severe depression, in primary care in India: a randomised controlled trial
Source: Lancet. 2017 Jan 14;389(10065):176–85. doi: 10.1016/S0140-6736(16)31589-6 (PMC5236064; doi:10.1016/S0140-6736(16)31589-6)
Supplement: Supplementary appendix [file mmc1.pdf]

THE LANCET

Supplementary appendix

This appendix formed part of the original submission and has been peer reviewed. We post it as supplied by the authors.

Supplement to: Patel V, Weobong B, Weiss HA, et al. The Healthy Activity Program (HAP), a lay counsellor-delivered brief psychological treatment for severe depression, in primary care in India: a randomised controlled trial. *Lancet* 2016; published online Dec 14. http://dx.doi.org/10.1016/S0140-6736(16)31589-6.

**Analysis**	**HAP arm (n=245)**	**EUC arm (n=248)**	**Adjusted mean difference or prevalence ratio (95% CI)**	**p-value**
**Sensitivity analyses**				
Complete case	(n=230)<br>19.88 (15.63)	(n=236)<br>27.27 (13.20)	-7.92 (-10.52, -5.33)	P<0.0001
Multiple imputation adjusting for counsellor as random effect	19.99 (15.70)	27.52 (13.26)	-7.97 (-11.26, -4.67)	P<0.0001
**Effect modification**				
**Baseline severity**			p-effect modification = 0.045	
Moderate	20.16 (15.82)	25.80 (13.19)	-6.01 (-9.14, -2.85)	p<0.0001
Severe	20.16 (15.45)	32.71 (12.75)	-12.11 (-17.83, -7.43)	P<0.0001
**Gender**			p-effect modification=0.70	
Males	20.44 (15.58)	29.26 (12.48)	-8.15 (-13.55, -2.75)	P=0.003
Females	19.90 (15.57)	27.13 (13.63)	-7.41 (-10.44, -4.39)	P<0.0001
**Baseline chronicity**			p-effect modification=0.91	
<12 weeks	17.94 (16.27)	25.32 (13.61)	-7.34 (-11.12, -3.55)	P<0.0001
>=12 weeks	22.43 (15.02)	30.46 (12.63)	-7.76 (-11.44, -4.09)	P<0.0001
**Expectation of counselling**			p-effect modification=0.42	
Not or somewhat useful	20.88 (16.56)	28.82 (11.91)	-7.78 (-11.65, -3.92)	P<0.0001
Moderate or very useful	19.37 (15.04)	26.51 (14.34)	-6.91 (-10.49, -3.35)	P<0.0001

Supplementary table 1: Additional analyses on primary outcome (BDI score at 3 months)

	**Participants (N=493)**	**Non-participants (N=290)**	**p-value**
**Age** (years) (mean [SD])	42.5 [12.0]	41.6 [12.2]	0.28
**Gender**			0.14
Male (n [%])	114 (67.9%)	54 (32.1%)	
Female (n [%])	379 (61.6%)	236 (38.4%)	
**Marital status** (n [%])			
Married	337 (61.6%)	210 (38.4%)	0.18
Single	57 (60.6%)	37 (39.4%)	
Separated/Divorced	95 (70.9%)	39 (29.1%)	
Widowed	4 (50%)	4 (50%)	
**Occupation** (n [%])			
Unemployed	292 (61.6%)	182 (38.4%)	0.33
Unskilled manual labour	174 (66.7%)	86 (33.1%)	
Skilled manual labour	7 (53.9%)	6 (46.2%)	
Clerical & professional	20 (55.6%)	16 (44.4%)	
**PHQ score** (mean [SD])	17.9 [2.7]	17.5 [2.6]	0.07
**PHQ score** (median [IQR])	17 (16-19)	17 (15-19)	0.08
**PHQ category** (n [%])			
Score 15-19 (Moderately severe)	372 (61.7%)	231 (38.3%)	0.18
Score 20-27 (severe)	121 (67.2%)	59 (32.8%)	

Supplementary table 2: Baseline characteristics of participants and non-participants

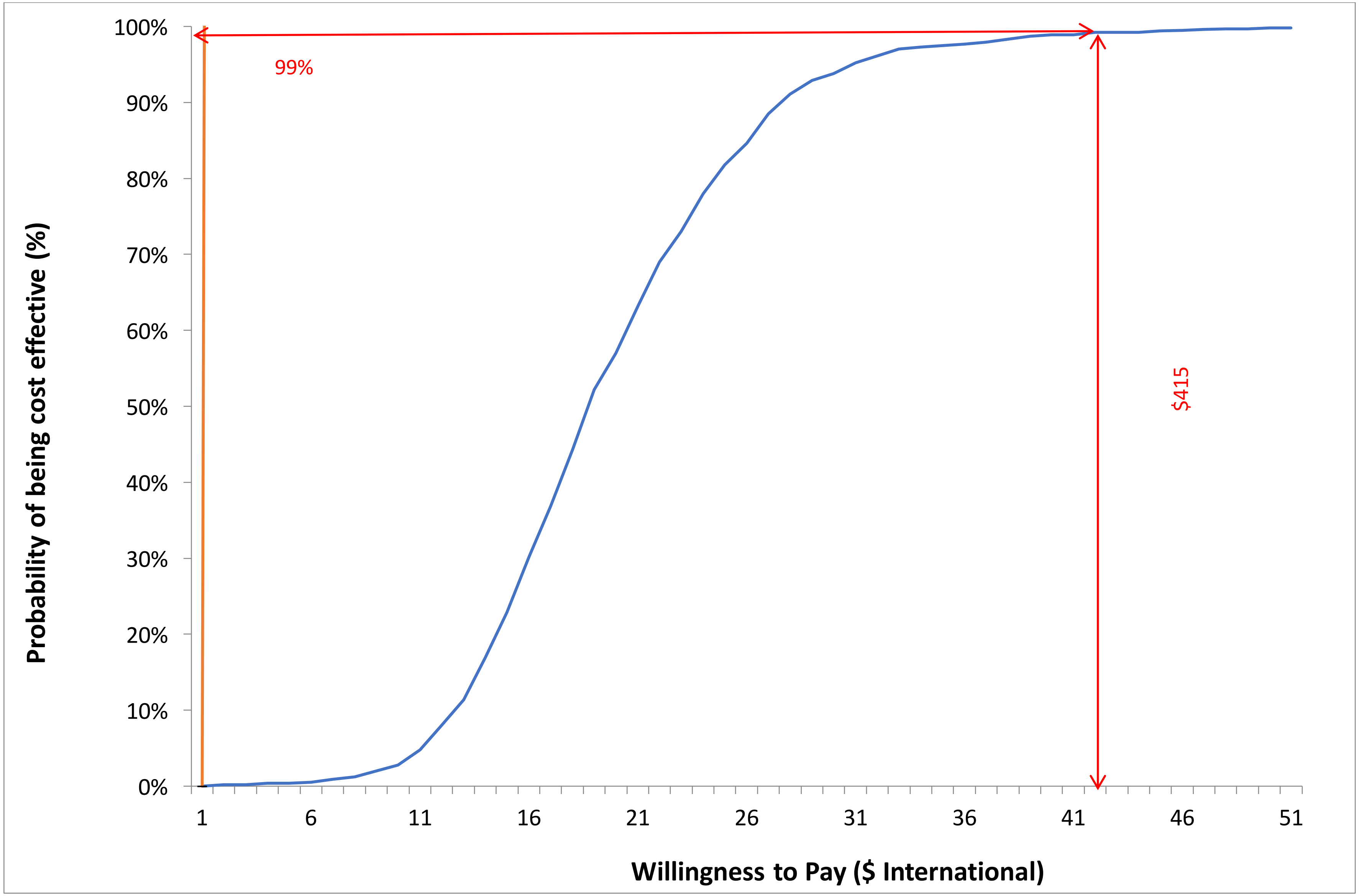
100%
90%
80%
70%
60%
50%
40%
30%
20%
10%
0%
99%
$415
1
6
11
16
21
26
31
36
41
46
51
Probability of being cost effective (%)
Willingness to Pay ($ International)

Supplementary figure 1: Figure: Cost-effectiveness acceptability curve: willingness to pay per remission achieved from the Healthy Activity Program from a health system perspective.

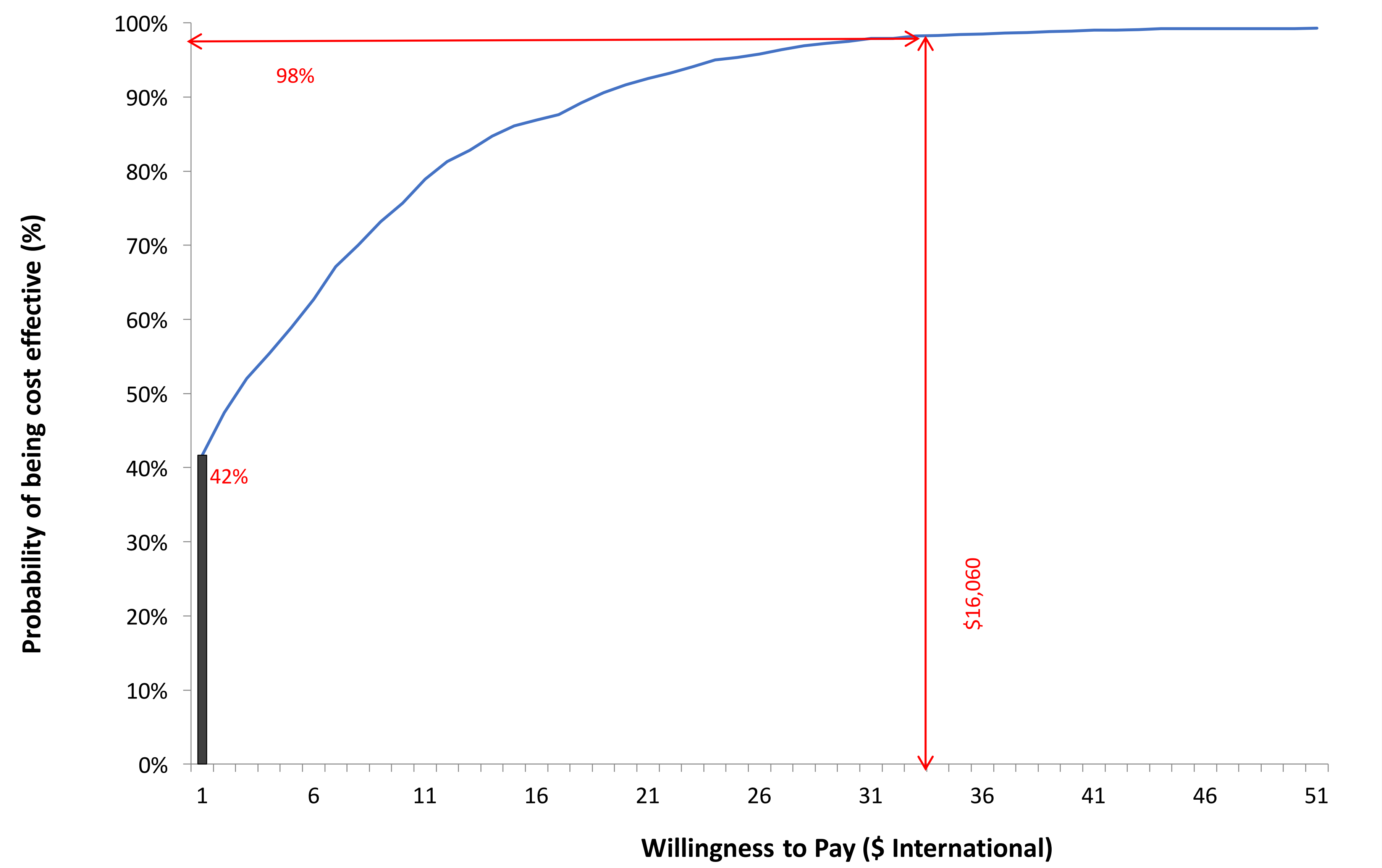

Supplementary figure 2: Figure: Cost-effectiveness acceptability curve: willingness to pay per QALY gained from HAP from a societal perspective.

Supplementary text 1: Search strategy

The search terms (in any field) included: Depression OR depressive
AND Psycho* OR therapy OR Counseling OR treatment. Concurrently, we carried out systematic reviews of regional (i.e. South Asian) literature on psychological treatments with searches of PubMed Central, IndMed, PsycINFO and PsycEXTRA (from 1 January 1990 to 1 January 2011). Search terms used were:
For PubMed, PsycINFo, PsycExtra:
• Depression OR Depressive OR anxiety
AND
• South Asia OR India OR Pakistan OR Bangladesh OR Sri Lanka OR Bhutan OR Nepal
OR Maldives OR Afghanistan
AND
• psycho* OR therapy OR counselling OR behaviour.
For IndMed:
1. Depression OR Depressive OR anxiety AND
2. Counseling OR psycho* OR therapy OR behaviour (each separately)
3. Psychotherapy
Search 1: (1) AND (2); Search 2: (3)

We also searched the ‘grey’ literature through hand searching reference lists of selected papers, contacting key informants and visits to key libraries in the region. Key findings were that while psychological treatment for depression was effective, most of the evidence was from specialist settings in high-income countries which limited its generalizability to primary care and low and middle income countries.

	Lost before 3 month evaluation (N=27)	Completed 3 month outcome evaluation (N=466)	p-value
**Age** (years) (mean [SD])	36.2 (11.6)	42.9 (12.0)	P<0.005
**Gender** (Female) (n [%])	23 (85%)	356 (76%)	P=0.36
**Marital status** (n [%])			
Married	16 (59%)	321 (69%)	P<0.006
Single	8 (30%)	49 (10%)	
Separated/Divorced	1 (4%)	3 (1%)	
Widowed	2 (7%)	93 (20%)	
**Education status** (n [%])			
None	6 (22%)	124 (27%)	P=0.71
Primary	13 (48%)	236 (50%)	
Secondary	5 (19%)	73 (16%)	
Higher Secondary	2 (7%)	22 (5%)	
Graduate/above	1 (4%)	11 (2%)	
**Occupation** (n [%])			
Unemployed	14 (52%)	278 (60%)	P=0.75
Unskilled manual labour	12 (41%)	162 (35%)	
Skilled manual labour	0 (0%)	7 (2%)	
Clerical & professional	1 (3%)	19 (4%)	
**Patient's expectation of counselling** (n [%])			
Not useful	0 (0%)	1 (0.2%)	P=0.24
A little/somewhat useful	8 (31%)	218 (47%)	
Moderately useful	8 (28%)	108 (23%)	
Very useful	11 (38%)	139 (30%)	
**Median PHQ score** (median [IQR])	17 (15-18)	17 (16-20)	P=0.36
**Mean PHQ-score (SD)**	17.3 (1.9)	18.0 (2.8)	P=0.20
**PHQ category** (n [%])			P=0.11
Score 15-19 (Mod. severe)	24 (90%)	348 (75%)	
Score 20-27 (severe)	3 (10%)	118 (25%)	

	Lost before 3 month evaluation (N=27)	Completed 3 month outcome evaluation (N=466)	p-value
**Trial Arm**			
**EUC**	12 (4.8%)	230 (93.9%)	P=0.53
**HAP**	15 (6.1%)	236 (95.2%)	

Web-table 1: comparison of participants who were followed up and LTFU at 3 months

**Variable**	**HAP** n participants (n events)	**EUC** n participants (n events)	**p-value**
*Any SAE	9 (9)	10 (10)	P=1.00
Death	2	0	P=0.24
Suicide attempt	4	3	P=0.72
Unplanned hospitalisation	3	7	P=0.34
Any psychotropic medication prescribed	23	23	P=0.91
Antidepressants prescribed	4	4	P=0.99

Web-table 2: Serious Adverse Events, and medication prescription, by trial arm (*number of trial participants with SAE=19)

	Health system perspective	Societal perspective
Cost per QALY gained at 3 months (95% CI)*	87%	98%
Cost per remission at 3 months (95% CI)**	99%	100%
Cost per BDI point improvement at 3 months (95% CI)***	100%	100%

Web-table 3: Likelihood of being cost effective for different outcomes and different perspectives
*Assumes willingness to pay threshold equivalent to GDP per capita in Goa ($16,060)
**Assumes willingness to pay threshold equivalent to one month's wages for unskilled manual worker in Goa ($415)
*** Assumes willingness to pay threshold equivalent to one day's wages for unskilled manual worker in Goa ($16.60)
